# Identification of a Protein-truncating Variant in *SCAPER* Gene Causing Syndromic form of Intellectual Disability

**DOI:** 10.2174/0109298673365248250312064016

**Published:** 2025-03-25

**Authors:** Monis Bilal Shamsi, Muhammad Zeeshan Ali, Safeer Ahmad, Mari Muurinen, Muzammil Ahmad Khan, Mohammed Turki Hussain Alharthi, Muhammad Latif

**Affiliations:** 1 Centre for Genetics and Inherited Diseases (CGID), Taibah University, Madinah, 42353, Saudi Arabia;; 2 Department Basic Medical Sciences, College of Medicine, Taibah University, Madinah, 42353, Saudi Arabia;; 3 Gomal Center of Biochemistry and Biotechnology, Gomal University, Dera Ismail Khan, Pakistan;; 4 Research Program for Clinical and Molecular Metabolism, Faculty of Medicine, University of Helsinki, Helsinki, Finland;; 5 Children’s Hospital, University of Helsinki and Helsinki University Hospital, Helsinki, Finland;; 6 Folkhälsan Research Center, Folkhälsan Institute of Genetics, Helsinki, Finland

**Keywords:** *SCAPER*, intellectual disability, retinitis pigmentosa, intellectual developmental disorder and retinitis pigmentosa, whole exome sequencing, protein modelling and docking

## Abstract

**Background:**

Intellectual disability (ID) is characterized by impairments in cognitive functioning and adaptive behavior. Globally, it affects 1-3% of the general population, with an increased prevalence in consanguineous families. It is a clinically heterogeneous disorder that can manifest as a variable phenotype. Intellectual developmental disorder and retinitis pigmentosa (IDDRP) is a rare syndrome in which patients present with both ID and retinitis pigmentosa.

**Aims and Objectives:**

This study examined a consanguineous family to identify disease-associated pathogenic mutations and elucidate their potential functional impact in patients with IDDRP.

**Methodology:**

Clinical assessment of the patients revealed characteristics consistent with both intellectual disability (ID) and retinitis pigmentosa. Individuals affected by IDDRP were subjected to whole exome sequencing (WES), and the identified candidate pathogenic variants were validated by Sanger sequencing. Computational analyses were conducted to evaluate the impact of these mutations on the protein structure and function.

**Results:**

WES identified a protein-truncating variant, c.2605A>T (p.Lys869Ter), in the S-phase cyclin A-associated protein in the endoplasmic reticulum (*SCAPER*) gene. *SCAPER* has previously been reported to cause IDDRP. *In silico* analyses revealed structural and interactional alterations in the *SCAPER* protein. This variant is novel in the Pakistani population and has not been previously reported. This variant exhibits an autosomal recessive mode of inheritance and segregates among the investigated affected and unaffected family members.

**Conclusion:**

The present study expands the spectrum of disease-causing variants in *SCAPER* and will contribute to a better understanding of the genetic etiology of IDDRP.

## 1 INTRODUCTION

Intellectual disability (ID) is a developmental disorder of the nervous system characterized by impairments in cognitive abilities and alterations in behavior. ID frequently coexists with other mental health conditions, including attention-deficit/hyperactivity disorder and autism spectrum disorder [1]. The prevalence of ID in the general population ranges from 1-3%, with developed nations exhibiting a prevalence of approximately 1-2%. However, in populations with high rates of consanguineous marriages, the incidence of ID is expected to be higher [2]. The ID conditions are sub-categorized into non-syndromic and syndromic ID. In non-syndromic ID, the patients are only suffering from cognitive impairment or developmental delay. While, syndromic intellectual disability is a multisystem disease that is characterized by cognitive impairment accompanied by additional abnormalities like eye disorder, skin disorder, skeletal disorder *etc* [3]. Nevertheless, the distinction between syndromic and non-syndromic ID can be challenging due to subtle, difficult-to-diagnose phenotypes [4]. Intellectual developmental disorder and retinitis pigmentosa (IDDRP) is a rare form of syndromic ID, featuring mild to moderate cognitive impairment and late-onset retinitis pigmentosa (typically manifesting in the second or third decade of life). The genetic etiology of this condition is a mutation in the *SCAPER* gene, which encodes the S-phase cyclin A-associated protein in the endoplasmic reticulum.

*SCAPER* (OMIM# 611611) gene contains 46 exons and is located on chromosome 15q24.3. It encodes a 158 kD S-phase cyclin A-associated protein, which contains a single zinc finger Cys2-His2 (C2H2 type), four coiled-coil regions, an endoplasmic reticulum (ER) retrieval signal at the C-terminal, and a putative transmembrane domain [5]. The RXL motif (cyclin binding motif) near the *N*-terminus of the *SCAPER* protein binds to cyclin A/Cdk2, and *SCAPER* acts as a regulatory protein that controls cell cycle progression [5, 6]. It has been observed that *SCAPER* protein and RNA levels are constant throughout the cell cycle but increase slightly during the S phase [5]. *SCAPER* protein is predominantly found in the ER; however, in minor amounts, it is also observed within the nucleus. Northern blot analysis has shown that *SCAPER* protein is ubiquitously expressed in several human tissues, with the highest expression in the testes and brain [5]. *SCAPER* protein exists in two isoforms (isoforms 1 and 2). RT-PCR analysis has shown the expression of isoform 1 in several human tissues, including the brain and retina, while expression of isoform 2 was not found in the brain. In a mouse model, temporal expression analysis of total RNA isolated from the brain and eye indicated that the *SCAPER* protein has equal and high expression in both types of tissues [5]. Mice with a *SCAPER* null mutation exhibit male infertility, lack of spermatogenesis, decreased testes size and weight, reduced female fertility, and abnormalities in ovarian follicles [7]. In humans, biallelic mutations in the *SCAPER* gene can cause intellectual disability disorders and retinitis pigmentosa (IDDRP OMIM# 618195).

Considering the limited understanding of the genetic etiology of IDDRP, this study was performed to characterize the genomic variants in individuals with IDDRP. Herein, we report a protein-truncating mutation c.2605A>T(p.Lys869Ter) of the *SCAPER* gene in an extended family ethnically from Pakistan, presenting IDDRP in six individuals. Furthermore, *in silico* functional analysis of the identified mutations predicted a drastic effect on the protein structure and interaction.

## 2 METHODOLOGY

Blood samples from the affected and healthy individuals of the family were collected, and genomic DNA was extracted using a commercially available kit (GeneJet, Genomic DNA extraction kit, Thermo Scientific, USA) according to standard laboratory protocols. This study was approved by the Institutional Ethics Committee of Gomal University, Dera Ismail Khan, Pakistan. Written informed consent was obtained from adult patients, whereas for patients under 18 years of age, consent was taken from legal guardians or parents for the publication of their data. A family pedigree was drawn to confirm the mode of inheritance of the disease and the degree of consanguinity among parents. Clinical features were documented by observing the apparent phenotypes and performance on the verbal and practical tasks. Retinal images of patients were captured with a fundus camera for retinal evaluation.

### 2.1 Genetic Analysis

To identify the genetic cause of the disease, Whole Exome Sequencing (WES) was conducted on two affected individuals (IV-1 and IV-8). The sequencing library was prepared utilizing the xGen Exome Research Panel v2.0 Kit (Integrated DNA Technologies, Coralville, Iowa, USA), and sequencing was performed on a NovaSeq 6000 (Illumina, San Diego, CA, USA). FASTQ data files were generated from base call files using “bcl2fastq v2.20.0.422” to produce sequence reads. These reads were subsequently aligned to the reference genome (GRCh37/hg19) using BWA-mem 0.7.17 (arXiv:1303.3997v2 [q-bio. GN]) to create BAM files. Variant calling was executed using GATK v.3.8 [8]. The final step involved employing “EVIDENCE 3 billion (South Korea)” for variant annotation and interpretation. Furthermore, WES data were annotated using wANNOVAR [9] and Variant Studio software (v3.0) and analyzed manually using bioinformatics tools. Genes associated with autosomal recessive ID and retinal dystrophies were analyzed using exome data. We targeted rare homozygous and compound heterozygous variants as the pedigree demonstrated a clear autosomal recessive inheritance pattern. In manual filtration, variants with minor allele frequency (MAF) ≤0.005 in gnomAD and Kaviar allele count of ≤10 were considered. Computational tools, including VarSome https://varsome.com/, MutationTaster 2021 https://www.genecascade.org/MutationTaster 2021/, and Sorting Intolerant From Tolerant (SIFT) (http://sift.bii.a-star.edu.sg/), were used to determine the biological pathogenicity of the variants. Segregation analysis of the identified pathogenic variant was performed by Sanger sequencing of two affected individuals (IV-8 and IV-11) and their parents (III-3 and III-4). The Primer3 web tool [10] was used to design primers for Sanger sequencing, and BioEdit (offline tool) and BLAT (online tool) were used for sequence analysis.

### 2.2 *In Silico* Functional Analysis

Protein structural modelling and protein-protein interactions were used to predict the functional consequences of the identified variants.

#### 2.2.1 Protein 3D Structure Prediction

Protein 3D structures of *SCAPER* wild-type and mutant proteins were predicted using I-TASSER, an online tool for predicting the 3D structure of a protein [9, 11]). The *SCAPER* wild-type and mutant proteins were superimposed, and their structural changes were observed using the offline tool Chimera (version 13.1.1) [12].

#### 2.2.2 Protein-protein Interactions

A string database was used to find the top ten close functional interactors of *SCAPER* protein [13], and then the ClusPro tool was used to perform docking of wild-type and mutant *SCAPER* with its topmost interacting protein [14]. Visualization of docking results was performed using the offline tool LigPlot^+^ [15].

## 3 RESULTS

### 3.1 General and Clinical Description

The studied family belonged to the Saraiki ethnicity of the Pakistani population. The family pedigree exhibited an autosomal recessive mode of inheritance pattern and consisted of four generations, with six affected individuals (IV-1, IV-3, IV-5, IV-8, IV-11, and IV-12) in the 4^th^ generation (Fig. **1A**). The clinical findings of the family indicated that the family was suffering from a rare syndromic condition, that is, IDDRP, in which the affected individuals had an intellectual disability (mild to moderate), and had retinitis pigmentosa. The patient had reduced night vision, visual acuity, and restricted visual field. Detailed clinical findings are listed in Table **1**. All affected individuals had normal skin, no dental anomalies, and no known defects in any visceral organs. Ophthalmic findings clearly showed retinal degeneration with prominent pigmentation on the fundus, indicating retinitis pigmentosa (Fig. **1B**).

### 3.2 Genetic Analysis

WES data analysis identified a homozygous variant NM_020843:c.2605A>T in exon 20 of the *SCAPER* gene in two affected individuals (IV-1 and IV-8). The identified single-base substitution produces a premature stop codon and causes protein truncation at position 869 (p.Lys869Ter). Protein truncation probably triggers the non-sense-mediated decay of defective mRNA, which is the most likely reason for the loss of functional integrity of *SCAPER* or abnormal protein production. The minor allele frequency (MAF) of the identified *SCAPER* variant was 0.00003746 in the global population and 0.00003844 in the South Asian population, with a total of nine heterozygotes. VarSome, MutationTaster, and SIFT predicted that this variant was pathogenic. Genotyping of the variant, performed by Sanger sequencing, confirmed the segregation of the identified pathogenic variants in the affected individuals of the family (Fig. **1C**).

### 3.3 Protein 3D Structure Prediction

3D structural predictions of wild-type and mutant *SCAPER* protein indicated that the mutant protein failed to superimpose over the wild-type protein, and the variant affected the folding pattern of the protein. Structural comparison revealed that the mutant *SCAPER* protein was only 3.34% identical to the wild- type protein (Fig. **2A**).

### 3.4 Protein-protein Docking

Analyses of protein-protein interactions between wild-type and mutant *SCAPER* proteins and their close interactor CCNA2 revealed differences in docking sites. The interaction patterns of wild-type and mutant proteins with close interactors exhibited variations in terms of bonding types and amino acid residues involved. The wild-type *SCAPER* interacted with CCNA2 through 11 bonds, comprising six hydrogen bonds and five salt bridges. In contrast, the mutant *SCAPER* formed 16 bonds with CCNA2, including 11 hydrogen bonds and five salt bridges. The amino acid residues participating in the docking of wild-type *SCAPER* and CCNA2 were Glu938, Gln1337, Glu863, Cys1341, Lys860, Lys1400, Glu857, Ser923, Glu885, and Arg907. For the mutant *SCAPER* and CCNA2 docking, the involved residues were Leu147, Lys131, Arg111, Thr289, Thr292, Gln152, Lys156, Lys252, Arg255, Ser254, Lys159, Glu155, Ala345, and His338 (Fig. **2B**).

## 4 DISCUSSION

In this study, we identified a novel homozygous variant NM_020843:c.2605A>T in exon 20 of the *SCAPER* gene in two IDDRP-affected individuals from a consanguineous family using WES. This variant has an autosomal recessive mode of inheritance and was confirmed by Sanger analysis segregating between the affected and non-affected members of the family.

Regarding the characteristic clinical features of IDDRP, the affected individuals in this study exhibited features critical to both intellectual disability and retinitis pigmentosa. All six individuals exhibited mild-to- moderate IQ and impaired speaking ability, which are representative of developmental delays. Ocular findings of reduced visual acuity restricted visual field, and impaired night vision, which are among the core features of IDDRP, was present in all affected individuals in our study. The patients investigated in this study were 22-32 years old at the time of the study. Congruent to other reported studies in which late-onset symptoms of retinitis pigmentosa (in the 20s and 30s) were observed in IDDRP, impaired night vision due to retinitis pigmentosa was also detected by us. A plausible explanation for the late-onset, as suggested by Jauregui *et al.* (2019), is that *SCAPER* does not play a role in the development of photoreceptors but rather in their function and/or maintenance; therefore, the retinal phenotype is presented later in life [16]. It is notable that ocular anomalies such as myopia, cataracts, strabismus, and glaucoma were less associated with IDDRP; therefore, of the six patients, only one patient (IV-3) was found to have strabismus. Other clinical characteristics, including bone deformity, muscular dystrophy, epileptic seizures, and visceral organ defects, although reported in lesser numbers in IDDRP patients with *SCAPER* mutations [17], were not observed in any of the affected individuals. This subtle interpersonal variability of less observed clinical features between IDDRP patients with *SCAPER* mutations could probably be due to the effect of other genetic and/or environmental factors that modify the phenotypic outcome of *SCAPER* mutations [6]. Mutations in *SCAPER* gene mainly cause IDDRP. Mice with a *SCAPER* null mutation exhibit male infertility, lack of spermatogenesis, decreased testes size and weight, reduced female fertility, and abnormalities in ovarian follicles [7]. The *SCAPER* protein predictably harbors an RXL motif at the N-terminus, a single zinc finger C2H2-type domain, an ER retrieval signal at the C-terminus, four coiled-coil domains, and a putative transmembrane domain. Interaction studies have found that *SCAPER* protein interacts with cyclin A and acts as a regulator during the G1/S and G2/M phases of the cell cycle [5]. Höpfler *et al.* recently identified the role of the C-terminal domain in the autoregulation of tubulin in cells [18].

The *SCAPER* gene mutation in disease etiology was first reported in 2011 as a result of genetic mapping in an Iranian family with ID syndrome. Subsequently, multiple studies have reported the association between ID and attention-deficit/hyperactivity disorder (ADHD) in patients suffering from autosomal recessive syndromic retinitis pigmentosa. Although Perea-Romero *et al.* (2021) identified a *SCAPER* gene mutation, they did not provide a detailed clinical description of the affected individuals [19]. In a recent publication by Sharkia *et al.*, 2024, a total of 37 cases of *SCAPER* gene mutations were reported [17]. Phenotype-genotype characterization across these studies has identified ID and RP as phenotypes associated with *SCAPER* syndrome. The protein-truncating mutation (NM_020843:c.2605A>T, p.Lys869Ter) identified in this study may probably induce nonsense-mediated decay of defective mRNA, potentially leading to a loss of functional integrity of the protein or the production of an abnormal protein as interpreted by protein-docking and other *in silico* tools used by us. Sharkia and co- workers also performed bioinformatics analysis on protein-truncating mutations and speculated that such kind of mutations either affect protein expression or alter folding patterns [17]. The *SCAPER* protein has been less studied, and so far, only two types of functions have been correlated with its domains. One interaction is established with cyclin A and CDK2 [5], and the others with ribosomal proteins and TTC5. These domains are considered to be involved in cell cycle regulation and tubulin autoregulation [18].

*In silico* 3D modelling of the *SCAPER* mutation (p.Lys869Ter) revealed significant structural distortion of the mutant protein. In addition, SCAPER-CCNA2 protein docking revealed different interaction patterns between the mutant and wild-type *SCAPER* proteins with its close interactor CCNA2. This protein is a member of the highly conserved cyclin family that regulates the cell cycle. CCNA2 (cyclin A2) protein activates cyclin-dependent kinase 2 and thus eventually promotes transition through G1/S and G2/M. Tsang *et al.* (2007) have suggested that *SCAPER* may act as cyclin A/Cdk2 regulatory protein that transiently maintains this kinase in the cytoplasm [5]. The *SCAPER* gene is broadly expressed in multiple tissues, including the brain and eyes, suggesting that mutations in this gene could impact not only the brain but also other tissues [6, 20, 21]. Individuals diagnosed with homozygous or compound heterozygous *SCAPER* gene mutations typically present with similar characteristics, primarily intellectual disability and retinitis pigmentosa.

However, Jauregui *et al.* 2019 reported an exception, wherein a patient manifested non-syndromic retinitis pigmentosa [16]. Some previously documented patients have demonstrated additional diverse features, including strabismus, obesity, attention deficit hyperactivity disorder, alopecia areata, dyspraxia, autism, various forms of facial dysmorphism, brachydactyly, and short stature [20-23]. All patients in the current study had a milder form of intellectual disability and retinitis pigmentosa that manifested in the second decade of life. Along with the ID and RP, two of the patients (IV-8 and IV-11) had unbalanced gait, as previously reported in a single patient [6]. Strabismus was observed in one patient, consistent with previous reports of five patients from two distinct families. Furthermore, one patient exhibited a prominent nose, a characteristic previously noted in two patients from a single family [6]. The updated mutational spectrum of the *SCAPER* gene and a clinical comparison of all reported patients with *SCAPER* mutations are presented in Fig. (**3**) and Table **2**. Collectively, our research and previously published findings indicate that ID and RP constitute the primary phenotypes associated with biallelic *SCAPER* mutations. However, patients may also manifest additional features with reduced penetrance. Further discoveries from future studies would help to elucidate the exact role of *SCAPER* mutations in the etiology of IDDRP and will undoubtedly provide a better understanding of the pathways and mechanisms underlying the effect of pathogenic mutations in disease pathology.

## CONCLUSION

The current study is the second to report *SCAPER* gene mutations in consanguineous Pakistani patients with the IDDRP phenotype. Most *SCAPER* gene mutations are non-sense mutations leading to protein truncation at different positions and loss of C-terminal domains, which could be the probable reason for inter-familial phenotypic variability. Our clinicopathological study confirms the body of evidence that ID and RP are the core phenotypes of *SCAPER* mutations.

## Figures and Tables

**Fig. (1):**
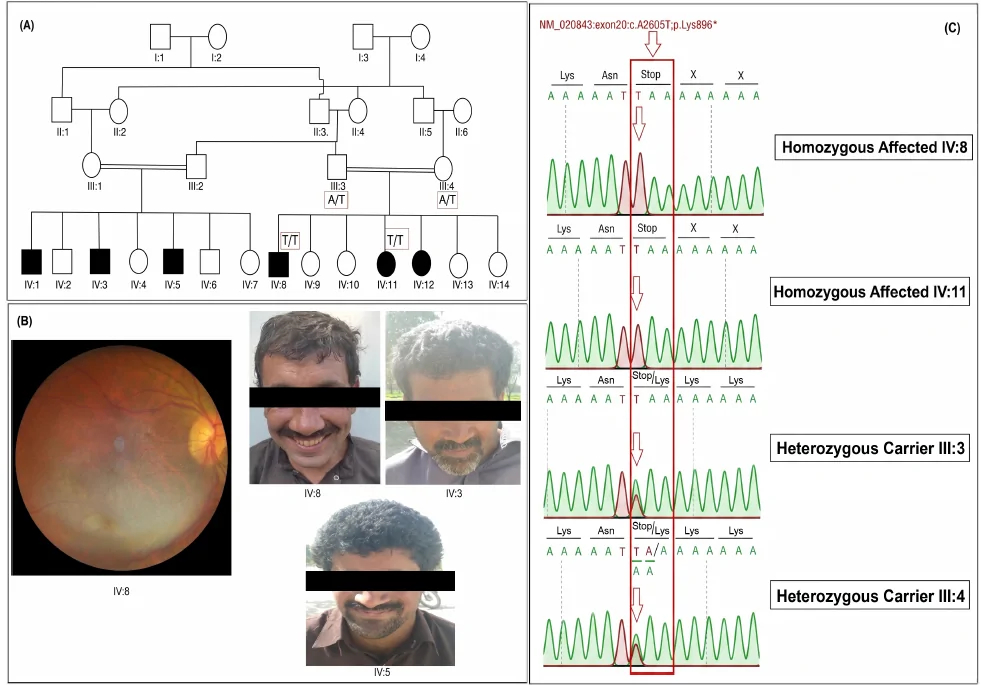
(**A**) Pedigree of the family showing autosomal recessive mode of inheritance. (**B**) Images of affected individuals of the family showing strabismus (IV-3) and broad nose (IV-5 and IV-8) and retina image of patient IV-8 showing pigmentation on the fundus. (**C**) Sequence chromatogram of affected individuals (IV-8 and IV-11) showing homozygous transversion mutation (A-->T), while both parents (III-3 and III-4) were revealing heterozygous/carrier genotype (A/T). Thus, the sequence eventually alters the Lysine codon to stop codon.

**Fig. (2):**
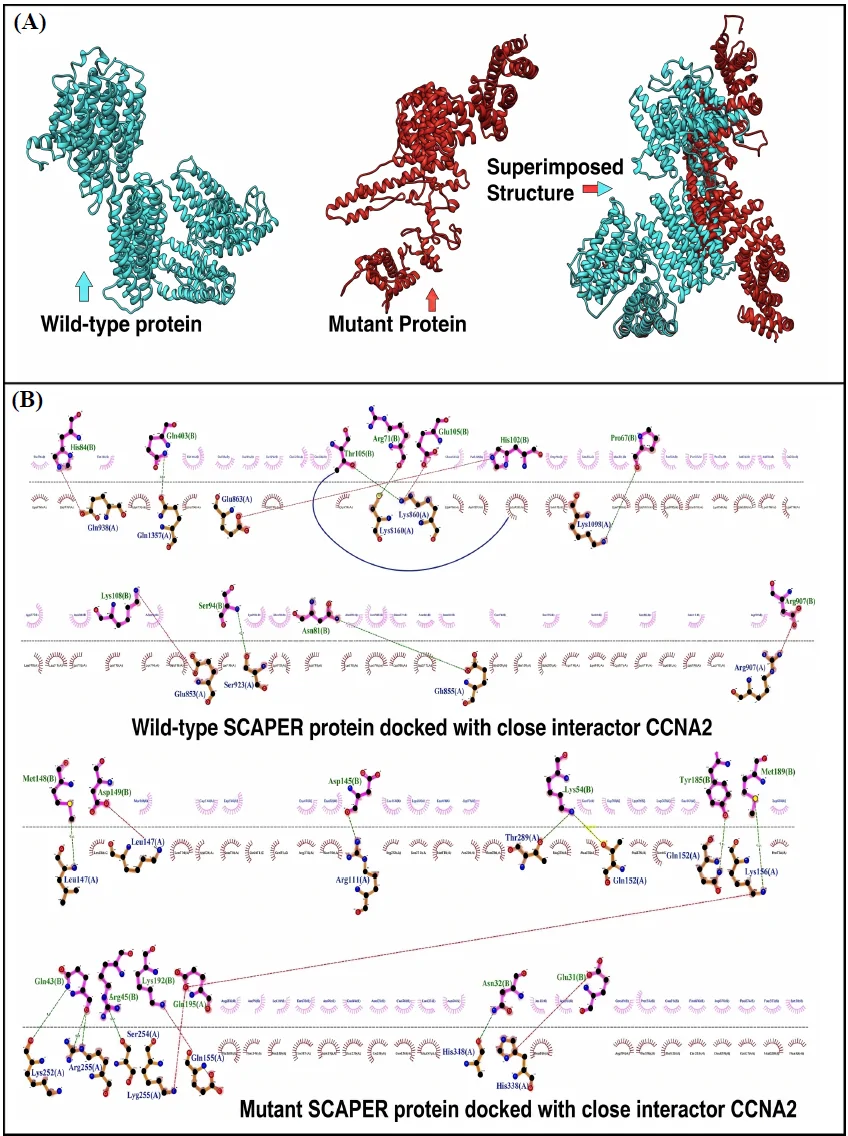
Protein 3D modeling and docking. (**A**) 3D structures of *SCAPER* wild, *SCAPER* mutant, and superimposed structure of *SCAPER* wild and mutant. (**B**) Molecular docking of *SCAPER* wild and mutant with close interacting protein CCNA2.

**Fig. (3):**
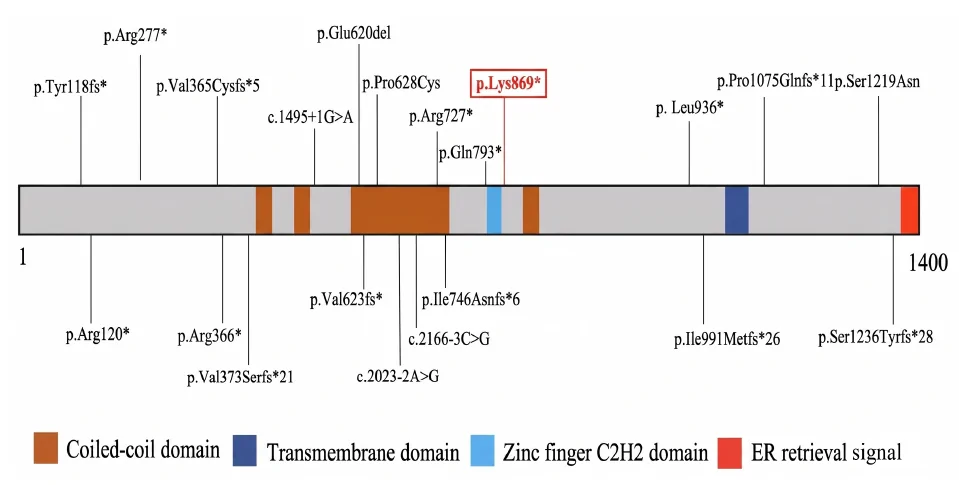
Schematic representation of *SCAPER* domains and the mutations it harbors til date. The mutation found in the current study is represented by red color above the protein structure. Note: * indicates the stop codons in the truncated protein, which are standard.

**Table 1:** Detailed clinical findings and phenotypes of the patients from current study.

**Phenotypic Features**	**Patient IDs**					
**-**	**IV-1**	**IV-3**	**IV-5**	**IV-8**	**IV-11**	**IV-12**
**Age (years)**	32	27	21	28	22	20
**Age of disease onset**	Congenital	Congenital	Congenital	Congenital	Congenital	Congenital
**Gender**	Male	Male	Male	Male	Female	Female
**IQ**	Mild to moderate	Mild to moderate	Mild to moderate	Mild to moderate	Mild to moderate	Mild to moderate
**Head Circumference**	Normal	Normal	Normal	Normal	Normal	Normal
**General Physique**	Normal	Normal	Normal	Normal	Normal	Normal
**Speaking ability**	Not fully developed	Not fully developed	Not fully developed	Not fully developed	Not fully developed	Not fully developed
**Night vision**	Impaired	Impaired	Impaired	Impaired	Impaired	Impaired
**Visual acuity**	Reduced	Reduced	Reduced	Reduced	Reduced	Reduced
**Visual field**	Restricted	Restricted	Restricted	Restricted	Restricted	Restricted
**Strabismus**	No	Yes	No	No	No	No
**Epilepsy**	No	No	No	No	No	No
**Bone deformity**	No	No	No	No	No	No
**Muscular dystrophy**	No	No	No	No	No	No
**Visceral organ defect**	No	No	No	No	No	No
**Movement of joints**	Normal	Normal	Normal	Normal	Normal	Normal
**Gait**	Wide gait	Normal	Normal	Wide gait	Normal	Normal
**Hearing ability**	Normal	Normal	Normal	Normal	Normal	Normal

**Table 2:** Mutation spectrum of *SCAPER* gene and phenotypic comparison of *SCAPER* mutations.

**Article**	**Family/Patients**		**Age**	**Gender**	**Genotype**	**Zygosity**	**Ethnicity/ Country**	**Major Phenotypes**	**Other Phenotypes**
(Najmabadi *et al.*, 2011)	Patient 1		NA	NA	p.Tyr118fs*	Hom	Iran	ID	NA
(Carss *et al.*, 2017)	Patient 1		NA	NA	p.Arg727*	Comp Het	NA	Retinal disease	NA
					p.Val373Serfs*21				
(Tatour *et al.*, 2017b)	Family A	Patient 1	24	F	c.2023-2A>G (E675-K677del)	Hom	Israeli Muslim Arab	ID+RP	ADHD
		Patient 2	23	F					
	Family B	Patient 1	34	F	p.Ile991Metfs*26	Hom	Spanish	ID+RP	Alopecia areata
	Family C	Patient 1	15	M	p.Glu620del	Comp Het	Spanish	ID+RP	Nil
					p.Ser1219Asn				
(Hu *et al.*, 2019)	Patient 1, 2, 3		NA	NA	p.Arg120*	Hom	Baloch	ID	NA
(Jauregui *et al.*, 2019)	Patient 1		11	M	c.2023-2A>G (same variant reported by Tatour *et al.* 2017)	Hom	Arab	NS-RP	Nil
(Wormser *et al.*, 2019)	Family A	Patient 1	34	F	p. Leu936*	Hom	Bedunion (southern Israel)	BBS (ID+RP) Note: Patient 4 of family B had RP suspected	[a]*
		Patient 2	28	M					
		Patient 3	24	M					
		Patient 4	17	M					
	Family B	Patient 1	48	F	p. Leu936*	Hom			
		Patient 2	47	F					
		Patient 3	29	F					
		Patient 4	10	M					
(Kahrizi *et al.*, 2019)	Family A	Patient 1	18	M	p.Val623fs*	Hom	Pakistani	ID+RP	[b]*
		Patient 2	12	F					[c]*
	Family B	Patient 1	34	F	p.Arg366*	Hom	NA	ID+RP	Prominent maxilla and micrognathia
		Patient 2	34	M					Strabismus, a prominent maxilla and micrognathia
		Patient 3	26	M					Prominent maxilla
	Family C	Patient 1	32	M	p.Val365Cysfs*5	Hom	NA	ID+RP	Strabismus, partial vitiligo on extremities
		Patient 2	12	F					Strabismus
		Patient 3	20	M					Strabismus
		Patient 4	25	M					Strabismus
	Family D	Patient 1	7	F	p.Pro628Cys	Hom	NA	ID+RP	[d]*
(Fasham *et al.*, 2019)	Family A	Patient 1	13	M	p.Ile746Asnfs*6	Hom	Amish	ID+(RP was not observed may be because of their age)	[e]*
		Patient 2	1.5	F					
	Family B	Patient 1 (Carss *et al.* 2017)	28	F	p.Val373Serfs*21	Comp Het	South Asian	Moderate ID+RP	ADHD, autism, self-harm
					p.Arg727*				
	Family C	Patient 1	31	F	c.1495+1G>A	Comp Het	Caucasian	Mild ID+RP	Dyspraxia
					p.Pro1075Glnfs*11				
	Family D	Patient 1	17	F	p.Arg277*	Comp Het	United States	ID+RP	Obesity
					p.Ser1236Tyrfs*28				
	Family E	Patient 1	24	F	p.Gln793*	Comp Het		Mild ID+RP	Moderate eczema with severe skin-picking behavior
					c.2166-3C>G				
Present Study	Patient 1		32	M	p.Lys869*	Hom	Pakistani	Moderate ID+RP	Unbalanced gait
	Patient 2		27	M					Strabismus
	Patient 3		21	M					Nil
	Patient 4		28	M					Unbalanced gait, Prominent nose
	Patient 5		22	F					Nil
	Patient 6		20	F					Nil

**Abbreviations:** ID, Intellectual disability; RP, Retinitis pigmentosa; Hom, Homozygous; Comp Het, Compound Heterozygous; M, Male; F, Female; ADHD, Attention deficit hyperactivity disorder; NS-RP, Non-syndromic retinitis pigmentosa; BBS, Bardet-Biedl syndrome; NA, not available; fs, frameshift. [a]* Obesity, Short stature, Brachydactyly, and Genu valgum/genu varum in all except patients 1 and 3 of family B; [b]* Polyneuropathy of the upper and lower limbs, global muscular hypotonia, high forehead and a narrow face with synophrys, a prominent nose, a symmetrical funnel chest and a syndactyly of the second and third toe on both sides; [c]* Hepatomegaly, asymmetry of the lateral ventricles, high forehead and a prominent nose; [d]* Slightly unbalanced gait, facial dysmorphism including slanting palpebral fissures, epicanthal fold, small-mouth, and low set ears; [e]* Proximally placed thumbs, short fifth fingers, pes planus, frontal bossing, almond-shaped eyes, and inverted nipples. Note: * indicates the stop codons in the truncated protein, which are standard.

## Data Availability

The data (sequence, photographs, and pedigrees) were stored in a password-protected computer at the Laboratory of Medical Genetics at Gomal University, D.I. Khan, Pakistan, and are available upon request.

## LIST OF ABBREVIATIONS

IDDRP: Intellectual Developmental Disorder and Retinitis Pigmentosa
ID: Intellectual Disability
WES: Whole Exome Sequencing
MAF: Minor Allele Frequency
SIFT: Sorting Intolerant From Tolerant
